# Impact of changes in systemic vascular resistance on a novel non-invasive continuous cardiac output measurement system based on pulse wave transit time: a report of two cases

**DOI:** 10.1007/s10877-013-9529-3

**Published:** 2013-11-07

**Authors:** Hironori Ishihara, Masato Tsutsui

**Affiliations:** 1 Department of Anesthesiology, Kuroishi-Kosei Hospital, Kuroishi-Shi, 036-0351 Japan; 2 Department of Anesthesiology, National Defense Medical College, 3-2 Namiki, Tokorozawa-Shi, 359-8513 Japan

**Keywords:** Cardiac output, Measurement technique, Pulse wave transit time, Systemic vascular resistance

## Abstract

The inaccuracy of arterial waveform analysis for measuring continuos cardiac output (CCO) associated with changes in systemic vascular resistance (SVR) has been well documented. A new non-invasive continuous cardiac output monitoring system (esCCO) mainly utilizing pulse wave transit time (PWTT) in place of arterial waveform analysis has been developed. However, the trending ability of esCCO to measure cardiac output during changes in SVR remains unclear. After a previous multicenter study on esCCO measurement, we retrospectively identified two cases in which apparent changes in SVR developed in a short period during data collection. In each case, the trending ability of esCCO to measure cardiac output and time component of PWTT were analyzed. Recorded data suggest that the time component of PWTT may have a significant impact on the accuracy of estimating stroke volume during changes in SVR. However, further prospective clinical studies are required to test this hypothesis.

## Introduction

**Fig. 1:**
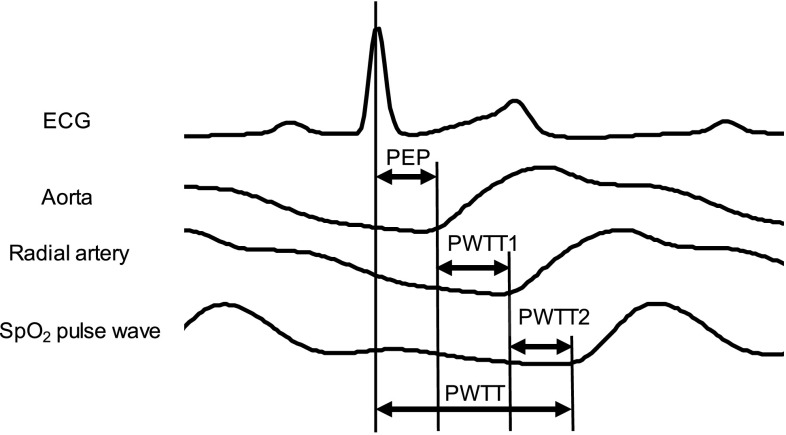
Relationship between each time-related component of pulse wave transit time. *PWTT* pulse wave transit time, *SpO*_*2*_ peripheral pulse oximetry, *PEP* pre-ejection period, *PWTT1* transit time from the rising point of the aortic root pressure wave to the rising point of the radial artery pressure wave in the systolic phase, *PWTT2* transit time from the rising point of the radial artery wave to the rising point of the SpO_2_ pulse wave in the systolic phase

Non-invasive continuous cardiac output (CCO) monitors have become popular in operating rooms and intensive care units (ICUs). However, the inaccuracy of arterial waveform analysis for measuring CCO associated with changes in systemic vascular resistance (SVR) has been well documented [1, 2]. Sugo et al. [3] developed a new non-invasive CCO monitoring system called estimated continuous cardiac output (esCCO). In the esCCO system, CCO is derived by means of a conventional electrocardiogram (ECG) monitor, peripheral pulse oximetry (SpO_2_) system, and arterial pulse pressure. The esCCO system uses pulse wave transit time (PWTT) in place of arterial waveform analysis. PWTT is the sum of the pre-ejection period (PEP) and the time taken for pulse wave to travel from the ascending aorta to the SpO_2_ probe site (Fig. 1). PWTT is calculated from the interval between the R wave peak of the ECG and the rise point of peripheral SpO_2_ pulse wave when the ECG and SpO_2_ are recorded simultaneously. Stroke volume (SV) is derived as follows:

$${\text{SV }} = {\text{ K }} \times \, (\alpha \times {\text{PWTT }} + \beta )$$

Here, *α* is an experimental constant. K and *β* are derived from PWTT, heart rate, pulse pressure, and cardiac output (CO) at the start of measurement for each patient. Accordingly, the esCCO system requires a continuous measurement of PWTT, but not MAP after the start of measurement to calculate CCO.

There have been no clinical studies on the esCCO system concerning CCO estimation during changes in SVR or on the impact of the time component of PWTT on the estimation of CO, even though changes in SVR would have a less impact on esCCO system compared to CCO measurement system with arterial waveform analysis [4]. In our previous multicenter studies [4–6], we identified two cases in which apparent changes in SVR developed in a short period of time during data collection. In this report, we retrospectively analyze the time component of peripheral wave transmission in these two cases, and examined the contribution of the time component of PWTT in determination of SV and CO.

## Case history

The esCCO system was connected to a conventional ECG monitor, radial artery pressure monitor, and SpO_2_ system. A CCO monitor derived data from a flow directional pulmonary artery catheter (Swan-Ganz CCOmbo CCO/SVO2, ref. 744HF75, Baxter Healthcare, Edwards Critical Care Division, Irvine, CA) and was connected to the esCCO system. SVR is calculated continuously from CO, mean arterial pressure (MAP), and CVP using a standard formula. Additionally, the amplitude of SpO_2_ pulse wave was measured continuously, as described by Murray et al. [7].

### Case 1

**Fig. 2:**
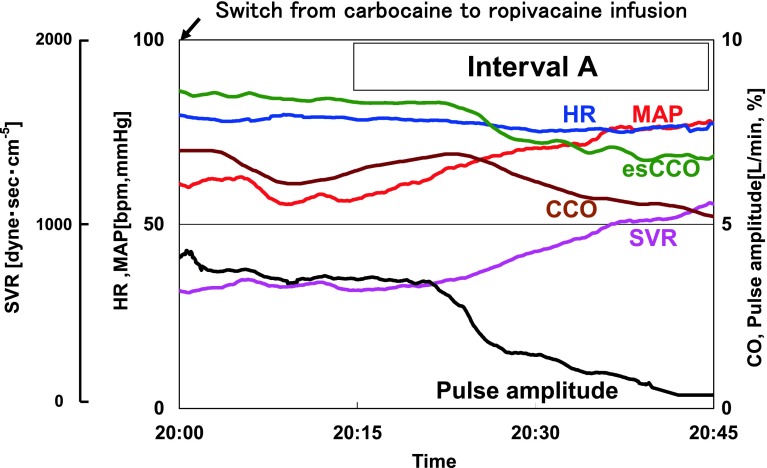
Trends in circulatory parameters in Case 1 (Interval A) *Blue* heart rate (HR); *Red* mean arterial pressure (MAP); *Green* estimated continuous cardiac output (esCCO); *Brown* continuous cardiac output derived from a flow directional pulmonary artery catheter (CCO); *Purple* systemic vascular resistance (SVR); *Black* pulse amplitude (normalized SpO_2_ pulse wave amplitude)

A 79-year-old male with height of 167.5 cm and preoperative body weight of 52.8 kg underwent removal of a pancreatic tumor. Total intravenous anesthesia with propofol and remifentanil combined with continuous epidural block using 6 ml/h of 1.0 % carbocaine was given to the patient. Approximately 2 h before completion of the surgical procedure, MAP started to gradually decrease and reached 61 mmHg at 20:00, and the carbocaine infusion was switched to 0.2 % ropivacaine infusion (5 ml/h). The lowest MAP of 55 mmHg was recorded at 20:09, and MAP gradually increased thereafter. While CCO decreased from 6.5 to 5.2 L/min (Fig. 2, Interval A: 20:15–20:45), SVR increased from 640 to 1,100 dyne s cm^−5^. esCCO decreased from 8.3 to 6.8 L/min, and the amplitude of the SpO_2_ pulse wave also decreased.

### Case 2

**Fig. 3:**
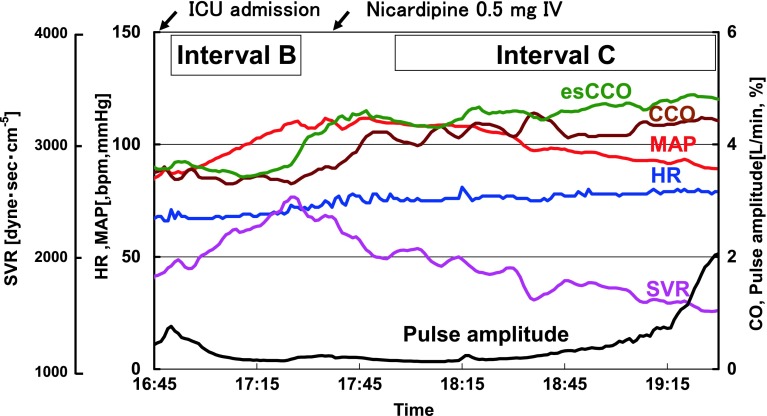
Trends in circulatory parameters in Case 2 (Intervals B and C) *Blue* heart rate (HR); *Red* mean arterial pressure (MAP); *Green* estimated continuous cardiac output (esCCO); *Brown* continuous cardiac output derived from a flow directional pulmonary artery catheter (CCO); *Purple* systemic vascular resistance (SVR); *Black* pulse amplitude (normalized SpO_2_ pulse wave amplitude)

A 61-year-old male with height of 158.5 cm and preoperative body weight of 56.9 kg underwent quadruple off-pump coronary artery bypass grafting. MAP increased, and SV slightly decreased immediately after the patient was admitted postoperatively to the ICU. SVR increased from 1,700 to 2,500 dyne s cm^−5^, but neither CCO nor esCCO was obviously changed (Fig. 3, Interval B). The amplitude of the SpO_2_ pulse wave decreased. Subsequently, 0.5 mg of IV nicardipine was injected and resulted in a decrease in MAP from 110 to 89 mmHg, associated with an increase in CCO from 4.0 to 4.5 L/min. SVR decreased from 2,500 to 1,600 dyne s cm^−5^. esCCO increased from 4.3 to 4.9 L/min, even though an increase in esCCO preceded that of the CCO, reflecting the time delay in CCO measurement due to averaging (Fig. 3, Interval C).

### Analysis of the time component

PWTT can be divided into the following 3 time-related components (Fig. 1):

1. PEP: Pre-ejection period consisting of the electromechanical delay at the start of systole and isometric contraction time, with the R wave of ECG serving as the starting point.
2. PWTT1: The time taken for pulse wave transmission from the aorta through the elastic arteries to the muscular arteries (namely, the radial artery).
3. PWTT2: The time taken for pulse wave transmission from the radial artery to the further distal peripheral site of SpO_2_ measurement.

As PEP and PWTT1 cannot be measured separately in the esCCO method, PWTT was divided into two components: PEP + PWTT1, and PWTT2.

**Fig. 4:**
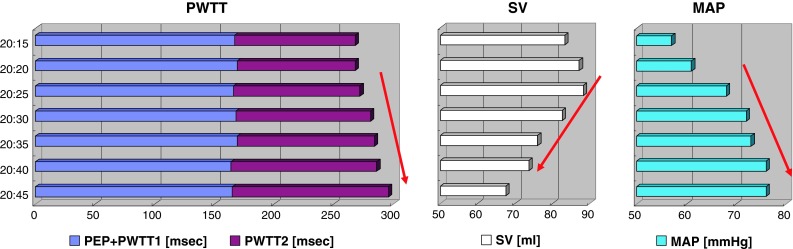
Trends in time-related components associated with stroke volume and mean arterial pressure from 20:15 to 20:45 in Case 1 (Interval A). *Left panel* PWTT (PEP + PWTT1 and PWTT2); *Middle panel* stroke volume (SV); *Right panel* MAP. *Bars* in each column show data obtained at 5-min intervals. *Red arrows* indicate directional changes over time

**Fig. 5:**
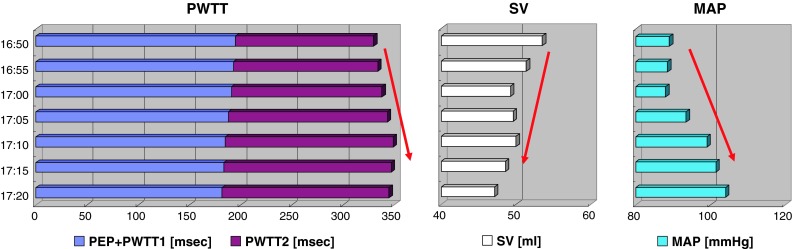
Trends of the time-related components associated with SV and MAP from 16:50 to 17:20 in Case 2 (Interval B). *Left panel* PWTT (PEP + PWTT1 and PWTT2); *Middle panel* SV; *Right panel* MAP. *Bars* in each column show data obtained at 5-min intervals. *Red arrows* indicate directional changes over time

When SVR increased in Case 1, PWTT increased in association with the decrease in SV and increase in MAP (Fig. 4). Among the components tested, PEP + PWTT1 remained unchanged, but PWTT2 increased during Interval A. A similar pattern of change was observed during the increase in SVR in Case 2 (Fig. 5, Interval B), even though PEP + PWTT1 tended to be slightly shorter.

**Fig. 6:**
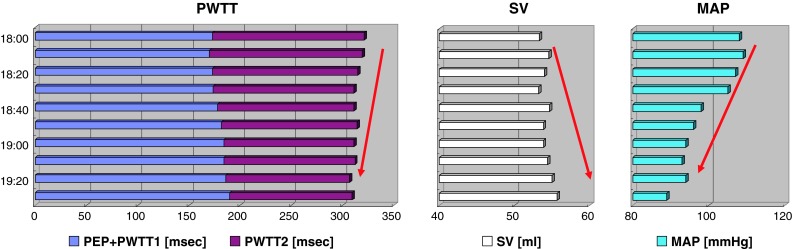
Trends in time-related components associated with SV and MAP from 18:00 to 19:30 in Case 2 (Interval C). *Left panel* PWTT (PEP + PWTT1 and PWTT2); *Middle panel* SV; *Right panel* MAP. *Bars* in each column show data obtained at 5-min intervals. *Red arrows* indicate directional changes over time

When SVR decreased in Case 2, PWTT tended to be shorter in association with the increasing tendency of SV and the decreasing tendency of MAP (Fig. 6, Interval C). PEP + PWTT1 showed a 1.10-fold prolongation at 19:30 compared with 18:00, and PWTT2 showed a 1.25-fold shortening at 19:30 compared with 18:00.

### Comment

Changes in SVR in both cases were confirmed by continuous estimation of SVR as well as by continuous measurement of the amplitude of the SpO_2_ pulse wave as reported by Murray et al. [7]. Additionally, contributory factors to induce changes in SVR were clearly identified in both cases: switch from carbocaine to ropivacaine infusion for case 1, and nicardipine injection for case 2, respectively. Therefore, we believe that this case report could preliminarily show the relationship between each component of PWTT and SV during changes in SVR.

Although PEP was not assessed separately in the esCCO system, PEP depends on changes in preload, afterload, and cardiac contractility. Ronn et al. [8] reported that PEP shortened when afterload was decreased by vasodilator administration. The inotropic action of the heart mediated by the carotid sinus reflex likely plays a role in this shortening. On the other hand, Boudoulas et al. [9] reported prolongation of PEP in relation to an elevation in isometric contraction pressure following an increase in afterload induced by vasoconstrictor administration. On the basis of these findings, we speculate that an increase in afterload prolongs PEP as observed in Case 1 (Fig. 4) and Case 2 (Fig. 5), while a decrease in afterload shortens PEP as observed in Case 2 (Fig. 6).

PWTT1 indicates pulse wave transit time from pulse waves arising from the aorta to the radial artery through elastic and muscular arteries. The pulse wave velocity of this time component is well known to correlate with arterial capacitance [10] and to be a useful parameter for measuring changes in arterial pressure [11]. When MAP increases, the pulse wave velocity increases, resulting in shortening of PWTT1 as observed in Case 1 (Fig. 4) and Case 2 (Fig. 5). In contrast, when MAP decreases, the pulse wave velocity decreases, resulting in prolongation of PWTT1 as observed in Case 2. When observing opposite directional changes in PEP and PWTT1 during changes in afterload, changes in PEP + PWTT1 are not obvious as observed in Case 1 (Fig. 4) and Case 2 (Figs. 5, 6), compared with changes shown when each component is solely used to estimate SV.

PWTT2 indicates pulse wave transit time from the radial artery to the further distal arterioles of the SpO_2_ detection site. Pulse wave velocity in this part begins to decrease and reflects an increase in viscosity as the vascular diameter decreases [12]. Accordingly, prolongation of PWTT2 can be expected when vasoconstriction occurs as observed in Case 1 (Fig. 4) and Case 2 (Fig. 5). In contrast, shortening of PWTT2 can be expected when vasodilation occurs as observed in Case 2 (Fig. 6).

Considering these directional changes in the time component of PWTT associated with changes in afterload, PWTT2 is likely to have a significant impact on the determination of PWTT during changes in afterload. Sugo et al. [3] analyzed the relationship of each of these components with SV in an animal study, and they found that inclusion of the PWTT2 component can improve the accuracy of SV estimation after phenylephrine injection. If SV is estimated solely from PWTT1, which reflects mainly arterial pressure [11], overestimation of SV can be expected during vasoconstriction accompanied by an increase in arterial pressure, while the underestimation during vasodilatation is associated with a decrease in arterial pressure. In fact, Meng et al. [2] reported that the trending ability of CO derived from arterial waveform analysis versus CO derived from esophageal Doppler was only 23 % after phenylephrine treatment, even though the former accurately tracked changes in CO when preload changed. It seems that the inclusion of PEP and PWTT2 can help promote the accuracy of estimating SV based on PWTT when changes in SVR occur.

There were limitations in this report in addition to a small sample size. First, data of this report were not derived from a prospective clinical study, and it is thus difficult to draw any definite conclusion, even though a canine experimental study on SV estimation after phenylephrine injection supported the hypothesis [3]. Therefore, further prospective clinical studies are required to test the hypothesis by simultaneously measuring MAP, CVP and well accepted CCO in comparison with esCCO. Second, the impact of changes in SVR on CO is only partially understood and consequently any conclusion based on simple observation is difficult. In fact, we could find only two cases where changes in SVR mainly affected changes in CO, since various factors other than afterload may also have a significant impact on determining CO particularly in our previous multi-center study [4].

In summary, data of this report suggest that the time component of PWTT may improve the accuracy of CCO estimation during changes in SVR. However, further prospective clinical studies are required to test this hypothesis.
